# Granular cell tumor of the breast: correlations between imaging and pathology findings

**DOI:** 10.1590/0100-3984.2019.0056

**Published:** 2020

**Authors:** Natacha Abreu, Juliana Filipe, Saudade André, José Carlos Marques

**Affiliations:** 1 Hospital Professor Doutor Fernando Fonseca EPE, Amadora, Lisboa, Portugal.; 2 Instituto Português de Oncologia de Lisboa Francisco Gentil (IPOLFG), Lisboa, Portugal.

**Keywords:** Granular cell tumor, Breast neoplasms, Schwann cells, Tumor de células granulares, Neoplasia da mama, Células de Schwann

## Abstract

**Objective:**

To review the imaging features of granular cell tumors of the breast (on mammography, ultrasound, and magnetic resonance imaging), establishing a pathological correlation, in order to familiarize radiologists with this entity and make them aware of the differential diagnoses, other than malignancy, of lesions with spiculated margins.

**Materials and Methods:**

We reviewed the medical records (from a clinical-pathology database and picture archiving and communication system) of five patients with a pathologically confirmed diagnosis of granular cell tumor of the breast, treated at the Portuguese Oncology Institute of Lisbon, in the city of Lisbon, Portugal, between January 2012 and December 2018.

**Results:**

All five tumors exhibited imaging features highly suggestive of malignancy (BI-RADS 5 lesions), namely spiculated margins, significant depth, and posterior acoustic shadowing (on ultrasound). One tumor showed a kinetic curve indicative of washout on magnetic resonance imaging, two were adherent to the pectoralis muscle, and one was accompanied by skin retraction. Pathology provided the definitive diagnosis in all cases.

**Conclusion:**

Granular cell tumors of the breast pose a diagnostic challenge because they can present with clinical and imaging features mimicking malignancy, and the diagnosis is therefore provided by pathology. Radiologists should be familiarized with this entity, so they can be aware of the fact that breast lesions with spiculated margins can be indicative of diagnoses other than malignancy.

## Abstract (pt)

**Objetivo:**

Rever as características de imagem dos tumores de células granulares da mama (mamográficas, ultrassonográficas e de ressonância magnética), estabelecendo uma correlação anatomopatológica, no intuito de proporcionar aos radiologistas uma familiarização com esta entidade e alertar para outros diagnósticos diferenciais de lesões espiculadas além das malignas.

**Materiais e Métodos:**

Consulta dos processos clínicos (base de dados clínica, anatomopatológica e sistema de comunicação e arquivamento de imagens) de doentes seguidos no Instituto Português de Oncologia de Lisboa, com diagnóstico anatomopatológico confirmado de tumor de células granulares da mama, de janeiro de 2012 a dezembro de 2018.

**Resultados:**

Todos os tumores exibiram características de imagem altamente sugestivas de malignidade (BI-RADS 5), nomeadamente espiculações, crescimento em profundidade e atenuação posterior (ultrassonografia), um mostrou um perfil cinético com washout na ressonância magnética, dois estavam aderentes ao músculo peitoral e um associava retração cutânea. O diagnóstico definitivo foi anatomopatológico.

**Conclusão:**

Os tumores de células granulares da mama constituem um desafio diagnóstico, pois podem apresentar características clínicas e de imagem que mimetizam malignidade, pelo que o diagnóstico é anatomopatológico. Os radiologistas devem estar familiarizados com esta entidade de forma a considerá-la nos diagnósticos diferenciais de lesões espiculadas, além das lesões malignas.

## INTRODUCTION

Granular cell tumors (GCTs) are typically benign neoplasms. They were first described in 1926 by Abrikossoff, who suggested that they had a myofibroblastic origin^(1)^. They are therefore sometimes referred to as Abrikossoff’s tumors. Although they can be solitary, multifocal GCTs reportedly occur in 18% of cases. They occur most commonly in the head, neck, chest wall, and arms, the tongue being the single most common anatomic site involved, and can be visceral, intradermal, or submucosal^(1,2)^. Up to 8.5% of all GCTs arise in the breast^(3)^, accounting for 0.1% of all cases of breast neoplasms^(4)^. Their relevance lies in their ability to mimic breast malignancies clinically and radiologically^(4)^. Malignant GCTs of the breast are extremely rare, only six cases having been reported in the literature. Given their rarity, most of the literature on GCT of the breast consists of small case-series or case reports^(4)^. The imaging documentation is scarce-especially in magnetic resonance imaging (MRI)-and no specific features have been established.

In this study, we review the clinical, pathological, and radiological characteristics of GCT of the breast, with a special focus on the imaging spectra, to acquaint radiologists with the visual aspects of this entity in order to avoid false-positive diagnoses of breast carcinoma. To that end, we evaluated five cases of GCT of the breast.

## MATERIALS AND METHODS

Data were obtained from the medical records of the Portuguese Oncology Institute of Lisbon, in the city of Lisbon, Portugal. We identified five cases of GCT of the breast treated between January 2012 and December 2018, the diagnosis having been confirmed pathologically in all five cases. For each case, hematoxylin-eosin-stained slides were reviewed by a breast pathologist, and immunohistochemical staining for S-100 protein was performed. The corresponding imaging information was retrieved from the local picture archiving and communicating system (IDS7; Sectra AB, Linköping, Sweden).

Digital mammography and tomosynthesis were performed in a digital mammography system (Mammomat Inspiration Prime; Siemens Healthcare, Erlangen, Germany). Ultrasound images were obtained with a 5.5-18 MHz transducer in an advanced ultrasound system (Acuson S2000; Siemens Healthcare). All MRI examinations were performed with the patient in the prone position, in a 1.5-T scanner (Intera; Philips Medical Systems, Andover, MA, USA) or a 3.0 T scanner (Ingenia; Philips Medical Systems), with a dedicated breast coil. The imaging protocol includes a localizing sequence followed by an axial diffusion-weighted imaging sequence (at b-values of 0 s/mm^2^ and 1000 s/mm^2^). That is in turn followed by T1-weighted three-dimensional dynamic enhanced T1-weighted high resolution isotropic volume examinations (repetition time/echo time: 3.8/1.98 ms), at baseline (pre-contrast), as well as at 1, 2, 3, 4, and 5 min after administration of a gadolinium-based contrast agent (Gadovist, 0.1 mL/kg; Bayer Schering Pharma AG, Berlin, Germany), with post-processing of axial subtracted images and sagittal reconstructions at 1 mm, plus an axial T2-weighted turbo spin-echo sequence (repetition time/echo time: 2500/90 ms), with axial and sagittal subtracted maximum intensity projection reconstructions.

## RESULTS

**Figure 1:**
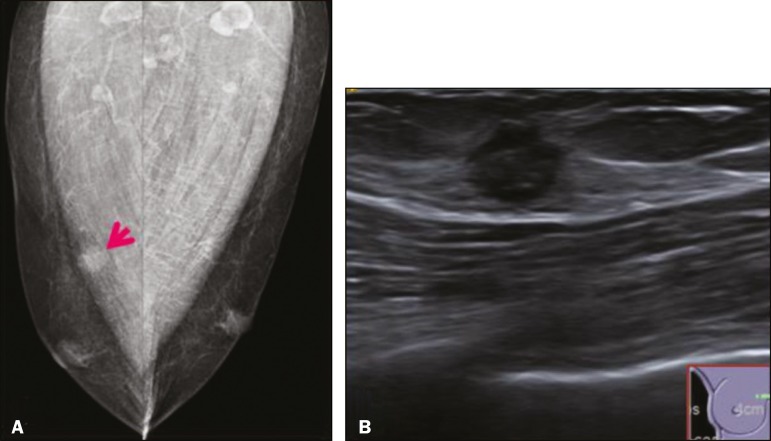
Digital mammography, in mediolateral oblique views (**A**), and ultrasound (**B**) in a 57-year-old male (case 1), showing a mass with indistinct margins in the upper inner quadrant of the right breast. The ultrasound shows a heteroechoic mass with microlobulated, fine spiculated margins, adjacent to the pectoralis major muscle. Pathology confirmed the diagnosis of GCT.

Four of the five cases reviewed occurred in women and one occurred in a male patient (Figure 1). All of the patients were White. One of the patients (a woman) was 20 years old, and the other patients were between 54 and 59 years of age. All of the GCTs presented as solitary nodules, in the left breast in three cases and in the right breast in two. Of the five GCTs, two were located in the upper inner quadrant, two were located in the upper outer quadrant, and one was located at the transition between the upper quadrants (Table 1). None of our cases presented with enlarged lymph nodes. All tumors were diagnosed by core biopsy, and the tumor was excised in all cases. At this writing, none of the tumors have recurred.

**Table 1:** Demographic and clinical characteristic of the five cases of GCT of the breast.

	Patient				Lesion				Imaging findings		
Case	Gender	Age (years)	Race		Location	Size (mm)	Depth (mm)		Digital mammography	Ultrasound	MRI
1	Male	57	White		R/UIQ	12	2		Mass; isodense; indis- tinct margins	Mass; spiculated margins; heteroechoic	-
2	Female	54	White		L/UIQ	28	3		Mass; hyperdense; circumscribed margins	Mass; irregular shape; heteroechoic; adjacent pectoralis; posterior acoustic shadowing	Irregular contours; T1 isoin- tense; T2 mildly hypoin- tense; type 1 kinetic curve
3	Female	20	White		L/UOQ	24	>10		Tomosynthesis; mass with gross spiculated margins	Mass; deep growth; spiculated margins; heteroechoic; posterior acoustic shadowing	Irregular contours; T1 isoin- tense; T2 mildly hypoin- tense; type 1 kinetic curve
4	Female	59	White		R/UOQ	12	15		Mass; isodense; spiculated margins; superficial	Mass; irregular contours; heteroechoic; subcutaneous; posterior acoustic shadowing	Spiculated margins; T1 isointense; T2 isointense; type 3 kinetic curve
5	Female	56	White		L7TUQ	13	1		Mass; mixed density; spiculated margins	Mass; deep growth; spiculated margins; heteroechoic; hyperechoic halo; poste- rior acoustic shadowing	-

R, right; L, left; UIQ, upper inner quadrant; UOQ, upper outer quadrant; TUQ, transition between the upper quadrants.

**Figure 2:**
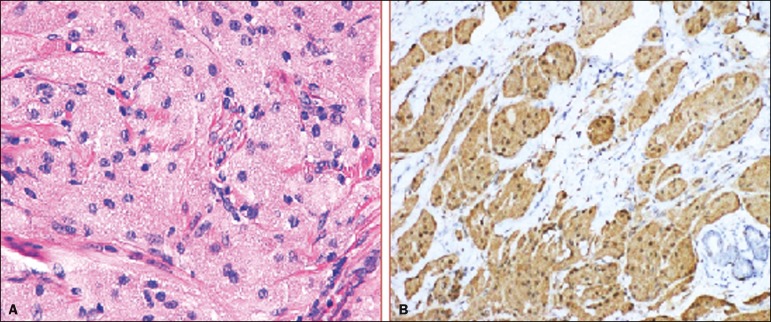
GCT specimen from a 57-year-old male (case 1). **A:** The cells have well-defined borders, round or oval nuclei with prominent nucleoli, and abundant granular cytoplasm, without atypia. **B:** Immunohistochemistry showing diffuse reactivity for S-100 protein.

Macroscopically, all of the GCTs were similar, presenting as firm, yellow masses with spiculated margins. Microscopically, they had a nested architecture, with large polygonal cells, abundant eosinophilic granular cytoplasm, and small, round to oval eccentrically located nuclei, with inconspicuous nucleoli (Figure 2A). There was no necrosis or evidence of mitotic activity. All the cases showed strong positive staining for S-100 protein (Figure 2B).

**Figure 3:**
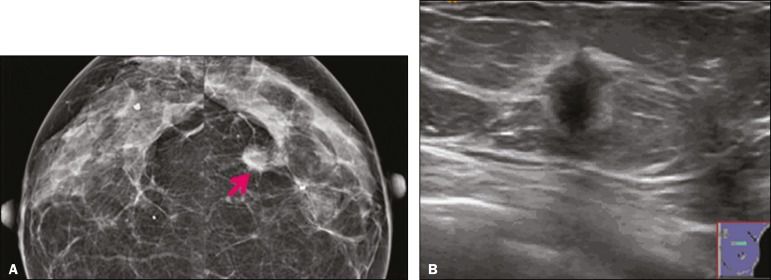
Digital mammography, in a craniocaudal view (**A**), ultrasound (**B**) in a 56-year-old woman (case 5) with GCT of the breast. Note the heterogeneous nodular density projecting into the transition between the upper quadrants of the left breast, with mixed density and some fine spiculated margins. In this heterogeneously dense parenchyma, the mass became visible because it protruded into the more adipose portion of the breast tissue. The mass was growing deep into the tissue and presented as an hypoechoic nodule (**B**) with an irregular shape and spiculated margins. It is surrounded by an hyperechoic halo, as described by Yang et al.^((20))^ as a possible consistent, typical finding of GTC of the breast.

**Figure 4:**
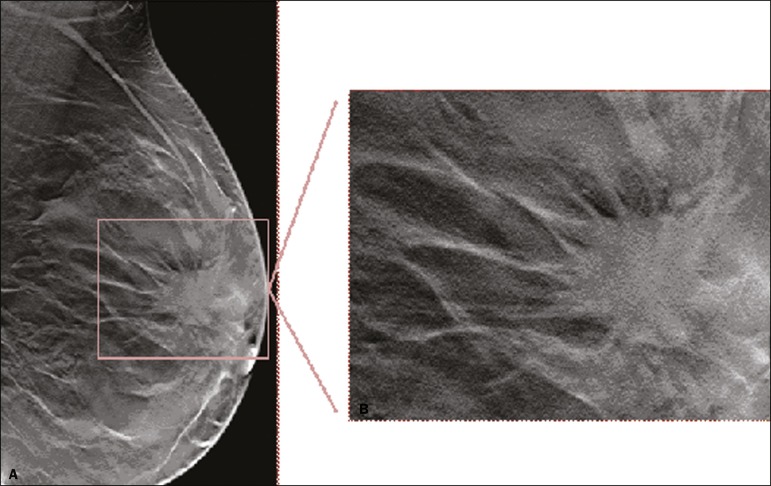
Tomosynthesis, in a mediolateral oblique view, of a 20-year-old woman (case 3)—same case as MRI shown in Figure 7—with GCT of the breast showing a mass with prominent spiculated margins, together with desmoplasia, which is highly suggestive of malignancy. Pathology confirmed the diagnosis of GCT.

**Figure 5:**
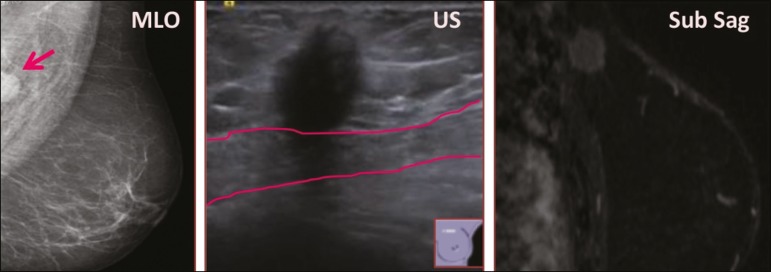
Digital mammography (mediolateral oblique view), ultrasound, and MRI in a 54-year-old woman (case 2) with a pathologically confirmed GCT in the upper inner quadrant of the left breast. The location of the mass was deep in the tissue, adjacent to the pectoralis muscle, which is partially intercepted in the mediolateral oblique view. However, it is so medially located that it is not intercepted in the craniocaudal view (not shown). On ultrasound, the mass showed suspicious features, such as nonparallel orientation (relative to the chest wall), microlobulated contours, and posterior acoustic shadowing. Note the close proximity to the pectoralis muscle (lined in pink), suggesting that the mass is adherent to it. The fine spiculated margins are best appreciated in the subtracted sagittal image.

**Figure 6:**
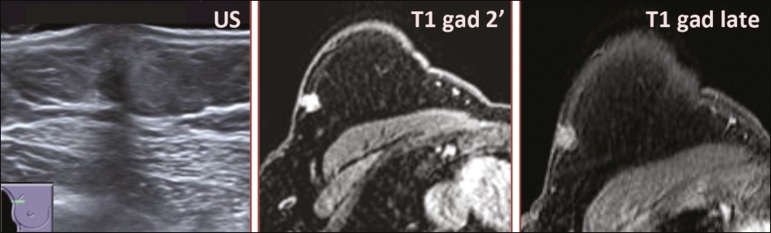
Ultrasound and MRI in a 59-year-old woman (case 4) presenting with a nodule in the upper outer quadrant of the right breast. The ultrasound shows that the mass is subcutaneous, a feature that has been recognized as a clue to the diagnosis of GCT. In gadolinium contrast-enhanced T1-weighted fat-saturated images, the nodule enhances avidly and shows washout. Note the fine spiculated margins. The morphologic features associated with this (type 3) kinetic curve would raise the suspicion of a malignant lesion. Pathology confirmed the diagnosis of GCT.

**Figure 7:**
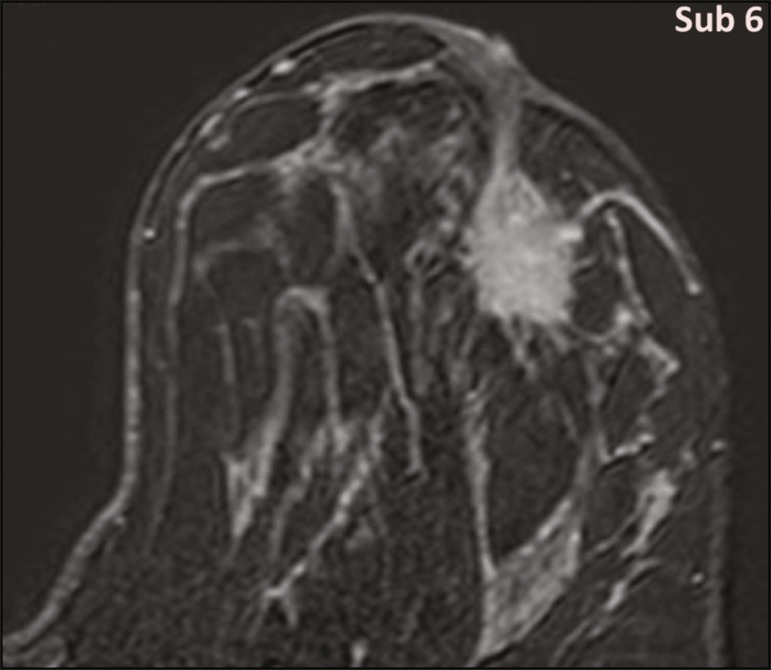
MRI of a 20-year-old woman (case 3) presenting with a mass in the upper outer quadrant of the left breast (delayed gadolinium enhancement—6 min—subtraction image). Note the skin retraction and spiculated margins, which suggest malignancy, although they essentially represent the desmoplastic reaction which is usually seen not only in carcinomas but also in GCT of the breast, as depicted in this pathologically confirmed GCT. The mass showed early mild enhancement with a type 1 kinetic curve.

The mammographic findings were nonspecific, all five of the lesions being characterized as masses (Figure 3), three exhibiting spiculated margins on mammogram (Figures 3 and 4), one showing circumscribed margins (Figure 5), and one showing indistinct margins (Figure 1). One GCT was located superficially, as demonstrated with ultrasound (Figure 6), whereas the others were located in different depths, two of them adjacent to the pectoralis fascia (Figure 5). Skin retraction was clearly noted in one case (Figure 7).

In all five of the GCTs evaluated, ultrasound showed nonparallel orientation (relative to the chest wall) and an irregular shape, both of which are highly suspicious features. On ultrasound, lesions with spiculated margins were observed in the same three cases documented in mammography. One GCT presented an hyperechoic halo (Figure 3B), and all five GCTs were heteroechoic.

Three of the five GCTs were examined with MRI, and all three presented as masses with no consistent imaging pattern among them. In T1-weighted sequences, they were all isointense to breast parenchyma, whereas they were either isointense or mildly hypointense to breast parenchyma in T2-weighted sequences. They all showed irregular, fine spiculated margins. The kinetic curves varied: of the three GCTs examined with MRI, one showed early, avid enhancement and subsequent washout (a type 3 curve), as depicted in Figure 6, and the other two showed mild progressive enhancement (type 1 curves).

## DISCUSSION

Although GCTs derive their name from the presence of abundant eosinophilic granules in the cytoplasm, their origin was inferred from an immunohistochemical finding (positive staining for S100 protein) and ultrastructural studies that revealed a neuroectodermal origin, most probably from Schwann cells^(3)^. They arise from the interlobular stroma, and some have theorized that they occurred within the distribution of the cutaneous branches of the supraclavicular nerve, namely the upper inner quadrant, which would substantiate the nervous origin of these neoplasms^(1)^. However, a wide variety of locations have been described^(5)^, as demonstrated in the present study. Granular-cell changes have also been found in association with mastectomy scars^(3,6)^. Although most reported cases of GCT have been in women, especially those who are premenopausal (despite the fact that the tumor is not hormone-dependent) or Black^(7)^, it can occur (rarely) in males^(8)^.

The presentation of a GCT is as a painless solid nodule that is usually firm on palpation and has a diameter of less than 3 cm. It is typically mobile but may be adhered to the pectoralis fascia. There can also be skin thickening, with or without retraction, and nipple inversion, clinical signs that mimic malignancy. It is the desmoplastic reaction usually invoked by these tumors that leads to retraction of the surrounding tissue and eventually the skin. That is responsible for their spiculated appearance on imaging, infiltrating pattern, and ill-defined margins^(9)^.

Most GCTs of the breast grow very slowly, stabilizing at approximately 3 cm in diameter. Any rapid growth in a lesion that has been stable for several years can suggest malignancy.

The radiological and histological assessment of a GCT is essential, and core biopsy specimens are usually sufficiently representative to provide the pathological diagnosis^(3,10,11)^. Rare cases of acinar cell-like breast carcinoma have been reported, and it may be considered in the differential diagnosis because it shows a diffuse infiltrative growth pattern of small glandular structures and is composed of cells with a granular or clear cytoplasm resembling acinar cells of the salivary glands. However, the immunohistochemical pattern differs from that of a GCT^(12,13)^.

Excision of a GCT of the breast is curative. However, because GCTs have an infiltrative growth pattern, wide surgical margins are required and have been shown to reduce the risk of recurrence^(14)^. Recurrence, not reported for any of the tumors evaluated in the present study, is reported in 2-8% of cases, even after excision with wide margins, although recurrence does not seem to affect the overall prognosis^(4)^.

Although GCTs can have a variety of presentations on mammography, most present with characteristics highly suggestive of malignancy, such as focal densities/masses with an irregular shape, blurred contours, and spiculated margins^(1)^, as was the case in all our patients, which challenged our diagnostic capability/accuracy. In some cases, a GCT presents with features usually associated with benignity (e.g., as a round mass with well-defined margins). Microcalcifications are not usually seen^(15)^. Because GCTs are often located subcutaneously, finding the epicenter of the lesion could provide a clue to the diagnosis, although they can be found at several depths, especially adjacent to the pectoralis muscle.

Ultrasound findings in GCTs are also nonspecific and typically favor a diagnosis of malignancy, especially when the mass is heteroechoic with spiculated, ill-defined margins and greater depth, with or without vascularization, particularly in the periphery^(16-18)^. Posterior attenuation reflects the amount of reactive fibrosis (desmoplastic reaction).

Some studies have suggested that the presence of sparse internal hyperechoic foci could be used to differentiate GCT of the breast from carcinomas, which are usually much more hypoechoic and homogeneous^(6,19)^. In addition, Yang et al.^(20)^ found a consistent hyperechoic halo or at least some hyperechoic component in five of their seven patients, suggesting that this could in part be related to the infiltrative growth pattern and also reflect their cellular origin (Schwann cells). However, none of these findings have been consistently reported in other studies. Although we also found this pattern in one of our patients, the other features of the nodule itself were highly suggestive of malignancy (spiculated margins and a vertical axis), which reasonably compelled us to perform a biopsy.

The MRI findings in GCT of the breast are also nonspecific, and the available image archive is limited. Hence, the features described to date rely on only a few reports, which are far from revealing a widely accepted set of findings. Although no consistent signal intensity has been observed in T1-weighted sequences, such GCTs are said to be isointense or hyperintense in T2-weighted sequences^(21,22)^. In T2-weighted sequences, GCTs seem to show less hyperintensity than do other masses, a finding that has been described as typical of GCT^(1)^. However, none of the GCTs evaluated in the present study showed hyperintensity in T2-weighted sequences. In fact, they were all isointense to hypointense in those sequences. In the literature, GCT of the breast is most frequently described as a round mass, with ill-defined, irregular, or spiculated margins, exhibiting a type 1 or type 2 kinetic curve, and has been reported to show ring enhancement in T2-weighted MRI sequences^(4)^.

Among the features usually associated with malignancy, only spiculated margins and ring enhancement have been described in GCT of the breast^(23)^. One of our cases showed a type 3 kinetic curve, a pattern which is not usually seen in benign lesions, having a specificity of 90.4%^(24)^, and which, taken together with the irregular morphology and spiculated margins of the lesion, was strongly indicative of malignancy. This case is particularly revealing about the ability of these tumors to mimic a carcinoma.

All in all, imaging findings do not present a consistent pattern. In suspected cases of GCT of the breast, MRI not only adds morphologic information but also helps determine the extent of disease, identify aggressive features, and screen the contralateral breast^(22)^. This highlights the important role played by imaging because, despite typically being solitary, GCTs of the breast may be multiple (in up to 18% of cases) within the affected breast, the contralateral breast, or elsewhere-such as in the context of Noonan syndrome with multiple lentigines^(3)^-as synchronous or metachronous lesions. The occurrence of metachronous lesions has divided authors, most considering them to be discrete primary lesions and others considering them to be “recurrences”, therefore recommending follow-up for at least ten years^(25)^.

There are no specific findings that allow a GCT diagnosis based on imaging features alone. Because the diagnosis depends on pathology, radiologists should not be absolutely confidant in a malignant etiology upon the finding of a lesion with spiculated margins and should consider the possibility of GCT of the breast despite its low incidence in comparison with that of breast carcinoma. Nevertheless, GCTs of the breast can present with coincidental carcinoma and some authors have described their occurrence in close proximity to (within 2 cm of) an invasive adenocarcinoma or as a collision tumor, with a carcinoma invading the GCT component^(25)^.

Malignant GCT of the breast is extremely rare, occurring in less than 1% of cases, there being only six cases reported in the literature^(26)^. Features suggestive of malignancy include a tumor diameter > 5 cm, cellular/nuclear pleomorphism, prominent nucleoli, a high level of mitotic activity, necrosis, and local recurrence^(3,27)^.

## CONCLUSION

It is known that GCT of the breast is a rare neoplasm that arises from Schwann cells. Its importance lies in its ability to mimic carcinoma clinically and radiologically. Although most GCTs of the breast are benign, 1% are malignant. Although there are no specific diagnostic imaging features, this entity should be borne in mind in the differential diagnosis of lesions with spiculated margins. The definitive diagnosis is made on the basis of the pathological characteristics, and tumor excision is curative. The prognosis of benign GCT of the breast is excellent, and local recurrence is uncommon.
